# Homogalacturonan From Yellow Passion Fruit Peel Ameliorates Intestinal Injury in DSS‐Induced Colitis in Mice

**DOI:** 10.1111/jcmm.70910

**Published:** 2025-10-30

**Authors:** Samilla Santos Souza Mazeti, Ariane Aviles Turini, Laryssa Regis Bueno, Cleiane Dias Lima, Ruan Sousa Bastos, Jefferson Almeida Rocha, Lucimara Mach Côrtes Cordeiro, Daniele Maria‐Ferreira, Marcelo Biondaro Gois

**Affiliations:** ^1^ Programa de Pós‐graduação em Biociências e Saúde Faculdade de Ciências da Saúde da Universidade Federal de Rondonópolis Rondonópolis MT Brazil; ^2^ Programa Institucional de Iniciação Científica (PIBIC), Graduanda em Medicina Faculdade de Ciências da Saúde da Universidade Federal de Rondonópolis Rondonópolis MT Brazil; ^3^ Programa de Pós‐graduação em Biotecnologia Aplicada à Saúde da Criança e do Adolescente, Faculdades Pequeno Príncipe Curitiba Brazil; ^4^ Instituto de Pesquisa Pelé Pequeno Príncipe Curitiba Brazil; ^5^ Grupo de Pesquisa em Química Medicinal e Biotecnologia (QUIMEBIO) Universidade Federal do Maranhão São Bernardo MA Brazil; ^6^ Programa de Pós‐Graduação em Biotecnologia (PPGBIOTEC) Universidade Federal do Delta do Parnaíba Parnaíba PI Brazil; ^7^ Programa de Pós‐graduação em Ciências – Bioquímica, Departamento de Bioquímica e Biologia Molecular Universidade Federal do Paraná Curitiba PR Brazil; ^8^ Programa de Pós‐graduação em Imunologia Instituto de Ciências da Saúde da Universidade Federal da Bahia Salvador BA Brazil

**Keywords:** colonic inflammation, in silico predictions, intestinal histoarchitecture, molecular docking, natural products, yellow passion fruit

## Abstract

Ulcerative colitis is a chronic inflammatory bowel disease characterised by persistent inflammation of the colonic mucosa. Symptoms include bloody diarrhoea and abdominal pain, which have a significant impact on quality of life. Current treatments mainly alleviate symptoms and maintain remission, but natural therapeutic alternatives are still needed. To investigate the therapeutic effect of pectic polysaccharides from the peel of *Passiflora edulis* on DSS‐induced ulcerative colitis in mice, focusing on colonic histoarchitecture, collagen remodelling, MUC‐2 expression and immune cell distribution. Mice were assigned to a control group, a group with DSS‐induced colitis, or a group with HPE treatment ( *P. edulis* homogalacturonan, 100 mg/kg). Histomorphometric, histopathologic, and immunohistochemical analyses of the colonic wall, evaluation of collagen remodelling and MUC‐2 expression, quantification of immune cells and in silico tests were performed. DSS caused atrophy of the muscular and submucosal layers, hypertrophy of the mucosa, increased crypt depth and decreased enterocyte height. HPE improved muscular and submucosal thickness, partially restored enterocyte height, reduced crypt depth and collagen deposition and increased intraepithelial lymphocytes. DSS significantly decreased MUC‐2 expression in goblet cells, while HPE significantly restored it. Pharmacokinetic and toxicological predictions indicated a favourable safety profile for HPE, and molecular docking analyses suggested interactions with proteins involved in maintaining the integrity of the intestinal barrier. HPE effectively attenuated DSS‐induced colon damage and restored MUC‐2 expression, contributing to epithelial barrier restoration and supporting its therapeutic potential in ulcerative colitis.

## 1 Introduction

Ulcerative colitis is a chronic inflammatory bowel disease characterised by continuous inflammation restricted to the colon and rectum [1], leading to progressive tissue damage and histoarchitectural remodelling. In contrast to Crohn's disease, which affects segmentally and transmurally any region of the gastrointestinal tract, ulcerative colitis affects the mucosa and leads to recurrent episodes of bloody diarrhoea, abdominal pain, tenesmus and weight loss [2]. The pathogenesis of ulcerative colitis is multifactorial and includes genetic predisposition and environmental factors [1], immunological dysfunction [3] and dysbiosis [4].

Clinical treatment of ulcerative colitis includes the use of corticosteroids, aminosalicylates, immunosuppressants and biologic drugs such as infliximab and vedolizumab, which are designed to reduce inflammation. However, the efficacy of these treatments is limited, with long‐term remission rates of less than 50% [1]. In addition, their side effects and high costs increase the need to explore new therapeutic approaches [5]. Alternative therapies based on natural products are therefore becoming increasingly important [6, 7]. Bioactive compounds extracted from plants, such as polysaccharides, have shown anti‐inflammatory and antioxidant properties in experimental models of ulcerative colitis, suggesting their potential as therapeutic adjuvants [7, 8, 9, 10, 11].

The morphological integrity of the colonic wall is essential for the maintenance of intestinal homeostasis. Loss of integrity, which is mainly characterised by increased intestinal permeability, is in turn devastating [7, 12]. Typical morphological changes in ulcerative colitis include muscle atrophy, collagen remodelling and distortion of the intestinal crypts [9], which impairs epithelial barrier function and allows translocation of microbial antigens, which in turn contributes to the maintenance of inflammation [13, 14]. Goblet cells, a type of epithelial cell, play a key role in this barrier by secreting the mucin MUC‐2, the main component of the mucus layer that separates the intestinal microbiota from the epithelium. Their reduction, which is frequently observed in ulcerative colitis, leads to decreased mucus production and further compromises the integrity of the epithelium, favouring dysbiosis and inflammation [4, 8, 9, 11]. As a direct consequence, ulcerative colitis causes damage to the enteric nervous system (ENS) [15], leading to motor [16] and sensory disturbances [17].

An important feature of ulcerative colitis is the disruption of the immune system, in which intraepithelial lymphocytes (IELs) and mast cells are involved. IELs, which have cytoprotective and immunoregulatory functions, are reduced in the inflamed mucosa, while mast cells, which are responsible for the release of inflammatory mediators, are increased and exacerbate the inflammation [18, 19]. The loss of regulatory IELs and the increase of cytotoxic IELs, which are activated by pro‐inflammatory cytokines such as IL‐23, play a central role in the pathogenesis of inflammatory bowel disease [20]. Activated mast cells secrete IL‐6 and TNF‐α, which promote the infiltration of eosinophils and IELs and intensify the inflammatory process [19].

Furthermore, the interaction between mast cells, IELs and enteric neurons suggests a direct link to the exacerbation of inflammation and the perception of pain in ulcerative colitis [21, 22]. This inflammatory cycle deregulates the synthesis and degradation of the extracellular matrix (ECM), leading to excessive accumulation of collagen in the intestinal wall. The increase in collagen, in turn, contributes to the development of fibrosis, impairs intestinal function and exacerbates the disease [23, 24].

In the present study, we investigated the therapeutic effects of pectin polysaccharides extracted from the peel of *P. edulis* f. *flavicarpa* (yellow passion fruit) in mice with dextran sulfate sodium (DSS)‐induced ulcerative colitis. The main objective was to evaluate the effects of the treatment on the maintenance of intestinal wall integrity, including colon wall layers, collagen remodelling, goblet cell maintenance and MUC‐2 expression and the distribution of immune cells such as IELs and mast cells. By exploiting these properties, we aim to contribute to the development of new effective, safe and affordable therapeutic strategies for the treatment of ulcerative colitis.

## 2 Methods

### 2.1 Ethical Aspects

Before starting the experiment, the protocol was submitted to and approved by the Ethics Committee for Animal Experimentation (CEUA) of the Pelé Pequeno Príncipe Research Institute, Curitiba, PR, under approval number 055‐2020. All procedures followed the ethical principles of the Brazilian Society of Laboratory Animal Science (SBCAL) and the ARRIVE (https://arriveguidelines.org/) guidelines.

### 2.2 Passion Fruit ( *P. edulis* ) Peel Pectin

The extraction and characterisation of polysaccharides from the peels of yellow passion fruit ( *P. edulis* f. *flavicarpa*), as described by Abboud et al. [25], involved several steps. In brief, the peels were obtained from fruits acquired in the local market (Curitiba–PR). After cleaning and separating the pulp, the peels were cut, freeze‐dried and ground into flour. The flour was defatted with hexane, and the soluble dietary fibre was extracted using the enzymatic–gravimetric method (AOAC 991.43) [25], yielding the fraction HPE.

Neutral monosaccharides in the polysaccharide fraction were analysed by gas chromatography–mass spectrometry (GC–MS) after derivatization, while the content of uronic acid was quantified by spectrophotometry [26]. High performance stereo‐exclusion chromatography (HPSEC), calibrated with dextran standards, was used to determine the molecular weight and homogeneity of the polysaccharides. The chemical structure of the isolated fraction was analysed by ^1^H, ^13^C and 2D‐^1^H‐^13^C HSQC nuclear magnetic resonance (NMR) spectroscopy.

The polysaccharide fraction (HPE, homogalacturonan from *P. edulis* ) consisted predominantly of galacturonic acid (92%), indicating the presence of highly methyl esterified homogalacturonan (DE = 70%), with minor amounts of arabinose, galactose and glucose [25].

### 2.3 Animals

Female Swiss mice (20–30 g) aged 4–5 weeks were provided by the Bioterium of the Carlos Chagas Institute, Fiocruz—Curitiba, PR. The animals were housed in plastic cages with wood shavings, with a maximum of 10 mice per cage. They were fed commercial food and had ad libitum access to water at a temperature of 25°C ± 2°C and a 12‐h light–dark cycle. The mice were acclimatised for 1 week prior to the experiment, and the bedding was changed every 3 days [8].

### 2.4 Induction and Evaluation of Ulcerative Colitis

**FIGURE 1:**
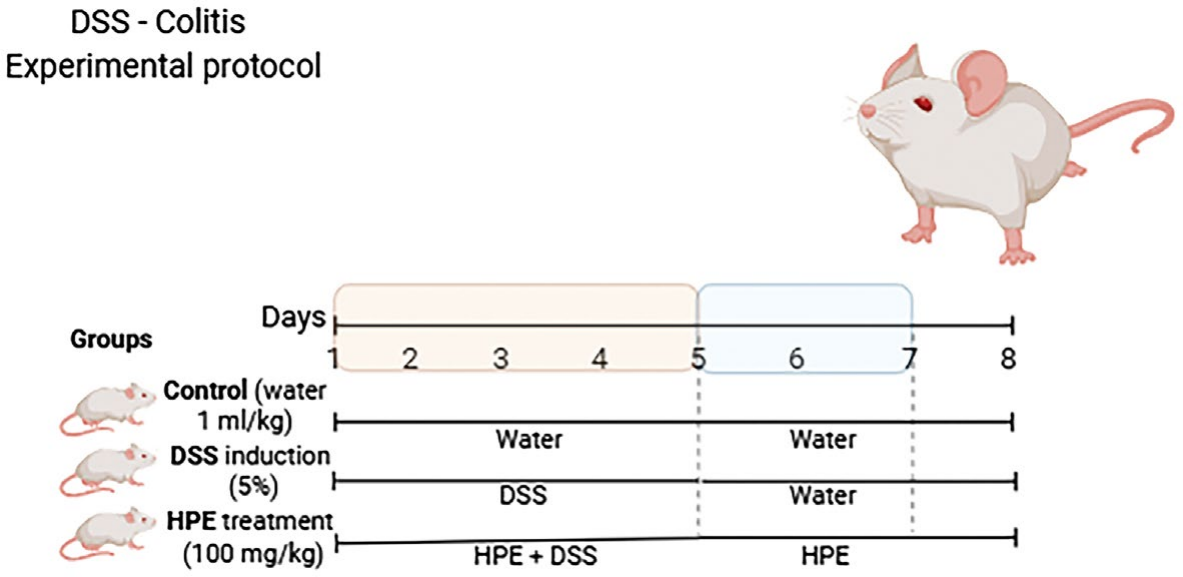
Induction of ulcerative colitis.

Acute ulcerative colitis was induced with 5% DSS (dextran sodium sulfate, molecular weight: 40,000, Cayman Chemical Company) in drinking water and administered on five consecutive days. On days 6, 7 and 8, the DSS was replaced with normal water [8, 9] (Figure 1).

### 2.5 Treatment and Disease Monitoring Protocol

Mice were randomly divided into three groups: (i) control (treated with vehicle), (ii) DSS (treated with vehicle and exposed to DSS) and (iii) HPE (treated with homogalacturonan extracted from *P. edulis* , 100 mg/kg per day for 7 days by gavage and exposed to DSS). Dose selection was based on previous studies. On day 8, all mice were euthanized by deep anaesthesia (lidocaine 4 mg/kg and thiopental 100 mg/kg), and colon samples were carefully collected, washed, and fixed in 4% paraformaldehyde buffered with PBS (pH 7.1) for 4 h [8, 25].

### 2.6 Histological Processing

Segments (1 cm) of colon were dehydrated in an ascending series of ethyl alcohol, diaphanized in xylene, and included in paraffin to obtain semiserial transverse sections of 5 μm, stained with haematoxylin and eosin (HE), Periodic Acid‐Schiff (PAS), Picro‐Sirius Red or Toluidine Blue (0.1%) staining to evaluate the histomorphometric, quantitative and histopathologic aspects of the colon wall [9, 27].

### 2.7 Histomorphometric Evaluation

The thickness (μm) of the muscle layer, the submucosa, the mucosa and the width and depth of the crypts were determined under 20× magnification in HE‐stained sections. For each parameter, 64 measurements were performed over the entire circumference of the colon of at least 6 mice per group. Images at 60× magnification were used to measure the height and width of 80 enterocytes. Histomorphometric analysis was performed using Image‐Pro Plus software (Media Cybernetics, USA) on images taken with a digital camera (Nikon DS‐Fi3 5.9 MP) connected to an optical microscope (Nikon Eclipse TS2R) [9, 27]. In addition, a qualitative histopathologic analysis was performed.

### 2.8 Morphological Assessment of the Myenteric Plexus

PAS‐stained sections were used for histomorphometric evaluation of the myenteric plexus. Ten ganglia from each mouse (six mice per group) were imaged using a microscope (Nikon Eclipse TS2R) equipped with a high‐resolution camera (Nikon DS‐Fi3 5.9 MP) and a 40× objective. The images were analyzed by measuring the area of the ganglia using Image‐Pro Plus software (Media Cybernetics, MD, USA). The result was expressed as the average area of the myenteric plexus ganglion profile in μm^2^ [11, 28, 29].

### 2.9 Evaluation of the Distribution of Intraepithelial Lymphocytes

The intraepithelial lymphocytes (IELs) were counted directly under the light microscope. Using the 40× objective, we counted IELs in a range of 2560 colonic epithelial cells of each mouse (640 epithelial cells/quadrant/section of at least 5 mice per group). For this purpose, we used HE‐stained sections to count the IELs. The number of IELs/100 epithelial cells was calculated [9, 28, 30].

### 2.10 Evaluation of the Mast Cells

Toluidine blue stained sections were used to quantify mast cells in the submucosa and mucosa of the colon. Mast cells were quantified blindly using a trinocular inverted microscope (Nikon Eclipse TS2R) with a 60× objective. Fifty random microscopic fields of the submucosa were counted for each mouse, with a minimum of five mice per group. The results were expressed as the average number of mast cells per microscopic field [31, 32].

### 2.11 Immunohistochemistry

The expression of MUC‐2 in the goblet cells of the colon was analysed by immunohistochemistry using a commercial kit (Cat. No.: E‐IR‐R217, Elabscience, China). Sections were deparaffinised, subjected to antigen retrieval in 10 mM citrate buffer (pH 6.0), and blocked with E‐IR‐R217C (3% H_2_O_2_) and non‐immune serum (E‐IR‐R217A, Normal Goat Blocking Buffer). They were then incubated overnight with a rabbit polyclonal antibody against MUC‐2 (Cat. No.: E‐AB‐70212, 1:200; Elabscience, China) or with antibody diluent alone as a negative control. After washing with PBS (pH 7.4; 8 × 5 min), sections were incubated with a secondary anti‐mouse/rabbit IgG antibody with polyperoxidase and visualised with 3,3′‐diaminobenzidine (DAB). Nuclei were counterstained with Mayer's haematoxylin, and sections were dehydrated, cleared in xylene, and mounted with Permount.

MUC‐2 expression was quantified in 10 images per animal (six animals per group) at 20× magnification. The intensity of staining was measured using ImageJ software and expressed as pixel values per microscopic field [8].

### 2.12 Evaluation of Collagen Remodelling

Sections were stained with Mallory trichrome to determine total collagen and with Picro‐Sirius red to determine type I and type III collagen of the colonic wall. To quantify collagen types, 32 images per mouse (from at least 5 mice per group) were captured using a 20× objective and a high‐resolution camera (Nikon DS‐Fi3 5.9 MP) connected to the optical microscope (Nikon Eclipse TS2R) and transferred to a microcomputer using image‐pro plus software (Media Cybernetics, MD, USA). Polarising filters (Olympus U‐POT Japan) were used to analyse the slides. Picro‐Sirius red staining shows that collagen I appears orange–red, while collagen III is coloured green. The results were expressed as a percentage of the amount of each collagen per area of the colonic wall [9].

### 2.13 In Silico Predictions

#### 2.13.1 Pharmacokinetic and Toxicological Predictions

The predictions of pharmacokinetic and toxicological properties (ADMET, absorption, distribution, metabolism and excretion) were performed using the pkCSM web server (http://biosig.unimelb.edu.au/pkcsm/) [33]. The structures of galacturonic acid were converted to SMILES format to perform prediction calculations for the pharmacokinetic profile.

#### 2.13.2 Molecular Docking

The software GaussView 5.0.81 and Gaussian 09w2 were used to create 3D structural models. Geometric optimization calculations were performed according to density functional theory (DFT) using the hybrid function B3LYP and basis set 6‐311G [34]. The ligand used was the monosaccharide galacturonic acid (α‐D‐Gal*p*A). The 3D structures of all targets were obtained from the Protein Data Bank (PDB) [35] with the codes 1XAW (crystal structure of the cytoplasmic distal C‐terminal domain of occludin) [36], 5Y2T (structure of the PPARgamma ligand‐binding domain‐lobeglitazone complex) [37], 6BSC (crystal structure of the mucin‐1 SEA domain) [38] and 6TM6 (MUC2 CysD1 domain) [39]. The Autodock 4.2 package was used for all docking procedures [40, 41]. The receptor was considered rigid, and each ligand was considered flexible. The partial charges according to Gasteiger were calculated after the addition of hydrogen atoms [42]. The non‐polar hydrogen atoms of the protein and the ligand were then combined. The region of the active site was determined using the FTMap server, which identifies the regions with the highest potential for ligand‐receptor interactions (ftmap.bu.edu/) [43]. The size of the cubic box was 40 × 40 × 40, and the *x*, *y* and *z* coordinates used can be seen in Table 1.

**TABLE 1:** Coordinates of the active sites obtained by FTMap.

Target	Coordinates of the grid centre (Angstrom)		
	*X*	*Y*	*Z*
1XAW	8.409	1.194	12.643
5Y2T	8.755	−0.616	47.506
6BSC	−16.428	7.088	6.842
6TM6	25.776	1.207	14.676

The Lamarckian global genetic search algorithm (LGA) [44] and the local search methods pseudo‐Solis and Wets (LS) were used for the docking search [45]. Each ligand was subjected to 100 independent docking simulation runs. Other docking parameters were set to default values.

### 2.14 Statistical Analysis

The D'Agostino‐Pearson test was used to assess the distribution of the data. For non‐normally distributed data, statistical analysis was performed using the Kruskal–Wallis test followed by the Dunn post hoc test, with results expressed as median and interquartile range. Statistical significance was set at *p* < 0.05. All analyses were performed using GraphPad Prism software (GraphPad Software). The specific statistical tests used in each analysis are indicated in the figure legends (^#^ *p* < 0.05 compared to the control group, *p < 0.05 compared to the DSS group, *n* = 6).

## 3 Results

### 3.1 Histomorphometric Analysis of the Colon Wall

To assess the extent of damage caused by DSS‐induced ulcerative colitis and the therapeutic effect of homogalacturonan (HPE) extracted from *P. edulis* in maintaining the integrity and histoarchitecture of the colon wall, we performed a detailed histomorphometric analysis. The results showed that DSS‐induced ulcerative colitis caused significant morphological changes in the colon wall, while treatment with HPE helped to alleviate some of this damage and support tissue recovery.

DSS‐induced ulcerative colitis caused significant atrophy of the muscle layer in the mice compared to the control group, with the median value decreasing from 132.1 μm (104.6; 158.7) in the control group to 43.1 μm (22.2; 98.1) in the DSS group (*p <* 0.05). In contrast, HPE treatment resulted in a significant improvement and promoted recovery and restoration of muscle layer thickness, with a median of 114.5 μm (72.6; 167.5) in the HPE group compared to the DSS group (*p <* 0.05; Table 2).

**TABLE 2:** Histomorphometric analysis of the layers composing the colon wall in mice with DSS‐induced ulcerative colitis (A).

Parameters (μm)		Control	DSS	HPE
Muscular	Thickness	132.1 (104.6; 158.7)	43.1 (22.2; 98.1)^#^	114.5 (72.6; 167.5)*
Submucosa		23.4 (14.9; 44.6)	10.8 (6.7; 19.1)^#^	37.1 (20.8; 53.2)*
Mucosa		158.8 (111.7; 215.5)	271.3 (203.7; 311.4)^#^	264.2 (167.4; 354.7)*
Crypts	Depth	85.4 (63.1; 114.4)	122.3 (73.6; 159.4)^#^	91.9 (62.7; 119.0)*
	Width	34.7 (30.1; 39.2)	34.8 (27.4; 41.5)	38.9 (32.6; 43.8)*
Enterocytes	Height	19.9 (17.1; 23.1)	16.2 (12.7; 20.5)^#^	18.1 (14.6; 21.1)*
	Width	8.2 (6.5; 10.2)	10.4 (8.5; 12.4)^#^	9.3 (7.7; 11.2)*

*Note:* Colitis was induced by administration of 5% DSS in drinking water for 5 consecutive days. Mice were treated orally with vehicle (control and DSS group: Water, 1 mL/kg) or 100 mg/kg of homogalacturonan extracted from *P. edulis* (HPE group) once daily for 7 days. Control: Control group, which received only water without DSS; DSS: DSS‐induced ulcerative colitis group, treated with vehicle (water) only; HPE: DSS‐induced ulcerative colitis group, treated with homogalacturonan extracted from *P. edulis* . Results were expressed as median with interquartile range (P25; P75) and analysed using the Kruskal–Wallis test followed by the Dunn post hoc test. ^#^ *p <* 0.05 compared with control group, *p < 0.05 compared with DSS group, *n* = 6.

As for the submucosa, mice with DSS‐induced ulcerative colitis showed significant atrophy in this layer compared to the control group, with the median value decreasing from 23.4 μm (14.9; 44.6) in the control group to 10.8 μm (6.7; 19.1) in the DSS group (*p <* 0.05). On the other hand, HPE treatment led to a significant hypertrophy of the submucosa with a median value of 37.1 μm (20.8; 53.2) in the HPE group compared to the DSS group (*p <* 0.05; Table 2).

Analysis of the colonic mucosa revealed that DSS‐induced ulcerative colitis caused significant hypertrophy of this layer compared to the control group, with the median value increasing from 158.8 μm (111.7; 215.5) in the control group to 271.3 μm (203.7; 311.4) in the DSS group (*p <* 0.05). Treatment with HPE resulted in a decrease in colonic mucosal thickness, with a median value of 264.2 μm (167.4; 354.7) in the HPE group compared to the DSS group (*p <* 0.05; Table 2).

Analysis of the histoarchitecture of the intestinal crypt showed that DSS‐induced ulcerative colitis causes a distortion of the depth (major axis) and width (minor axis) of the crypt. In mice with DSS‐induced ulcerative colitis, there was a significant increase in crypt depth compared to the control group, with the median value increasing from 85.4 μm (63.1; 114.4) in the control group to 122.3 μm (73.6; 159.4) in the DSS group (*p <* 0.05). In contrast, crypt width did not show significant change in the DSS group (*p* > 0.05). HPE treatment resulted in a significant decrease in the depth of the crypt with a median of 91.9 μm (62.7; 119.0) in the HPE group compared to the DSS group (*p <* 0.05). In addition, HPE treatment significantly increased the width of crypts in the colonic mucosa, with a median of 38.9 μm (32.6; 43.8) in the HPE group compared to the DSS group, which had a median of 34.8 μm (27.4; 41.5) (*p <* 0.05; Table 2).

To assess the extent of damage caused by DSS‐induced ulcerative colitis and the therapeutic effect of HPE on the colonic epithelium of mice, we performed a histomorphometric analysis of enterocytes. We found that both DSS‐induced ulcerative colitis and HPE treatment caused significant changes in the height (major axis) and width (minor axis) of enterocytes.

There was a significant decrease in the height of enterocytes in the mucosal epithelium of mice in the DSS group compared to the control group, with the median decreasing from 19.9 μm (17.1; 23.1) in the control group to 16.2 μm (12.7; 20.5) in the DSS group (*p <* 0.05). However, treatment with HPE resulted in a significant increase in enterocyte height, with a median value of 18.1 μm (14.6; 21.1) in the HPE group compared to the DSS group (*p <* 0.05). In terms of enterocyte width, DSS‐induced ulcerative colitis led to a significant increase in this parameter, with the median value increasing from 8.2 μm (6.5; 10.2) in the control group to 10.4 μm (8.5; 12.4) in the DSS group (*p <* 0.05). However, HPE treatment significantly decreased enterocyte width, with a median of 9.3 μm (7.7; 11.2) in the HPE group compared to the DSS group (*p <* 0.05; Table 2).

### 3.2 Qualitative Histopathological Analysis of the Colon Wall

**FIGURE 2:**
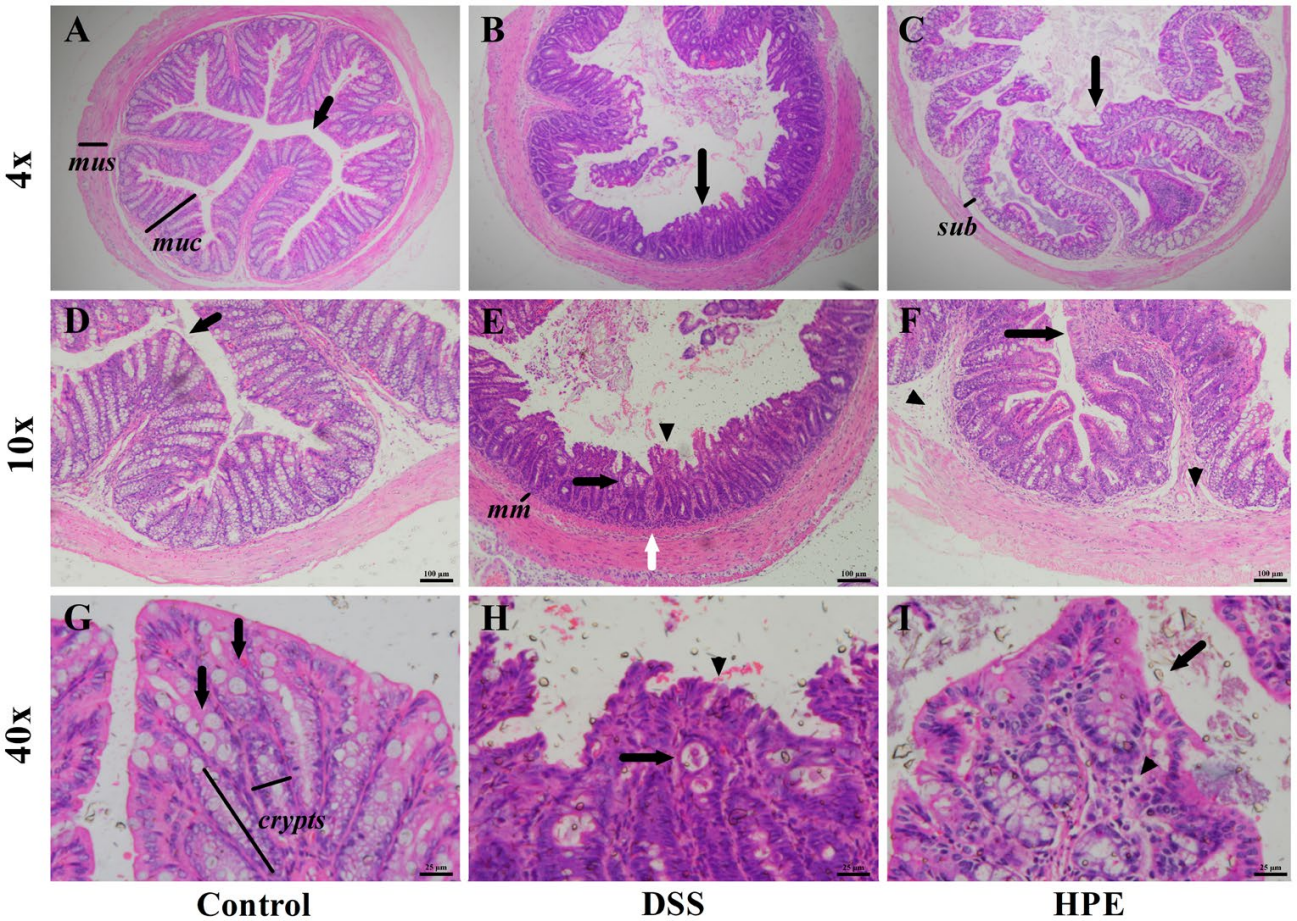
Representative photomicrographs of HE‐stained colon sections from control (A, D, G), DSS (B, E, H), and HPE‐treated mice (C, F, I). Ulcerative colitis was induced by administration of 5% DSS in drinking water for 5 consecutive days. Mice were treated orally with vehicle (control and DSS group: Water, 1 mL/kg) or 100 mg/kg homogalacturonan from *P. edulis* (HPE group) once daily for 7 days. Panels A–C were captured at 4× magnification, D–F at 10×, and G–I at 40×. The images are representative fields at each magnification, not enlargements of the same microscopic field. (A) The black arrow indicates a colonic fold representing normal colonic mucosa in the control group. (B) The black arrow indicates severely altered colonic mucosa characterised by hyperplasia, distortion of intestinal crypts, ruptures and loss of epithelial surface, indicating loss of colonic histoarchitecture. (C) The black arrow shows moderately altered colonic histoarchitecture with diffuse inflammatory infiltrate and increased submucosal thickness. (D, G) The arrows indicate the white areas corresponding to the goblet cells (negative) in the unchanged colonic mucosa. (E, H) Black arrows indicate abscess formation, arrowheads indicate epithelial rupture and erosions, and white arrows indicate diffuse inflammatory infiltrates in the lamina propria, submucosa and muscularis. (F, I) Arrows indicate ulcerations and epithelial erosions, while arrowheads show intense diffuse inflammatory infiltrates in the lamina propria and submucosa, primarily consisting of neutrophils and lymphocytes. Note the absence of white areas corresponding to goblet cells, suggesting their depletion. Calibration bars and the objective used for image acquisition are indicated in each image. Mus: Muscular; muc: Mucosa (A); sub: Submucosa (C); mm: Muscularis mucosae (E); and the major axis (depth) and minor axis (width) of the intestinal crypts are represented by the bars (G).

Qualitative histopathologic analysis revealed several changes in the colon wall of mice with DSS‐induced ulcerative colitis (Figure 2A–I). Intense inflammation, disruption of the epithelial barrier, hyperplasia and tissue remodelling were observed. The main feature was the infiltration of inflammatory cells, mainly neutrophils and lymphocytes, predominantly in the lamina propria. In some sections, involvement of the submucosa and muscle layer was also seen (Figure 2B,E,H). The observed thickening of the mucosa (Table 2) suggests that the infiltration led to edema and the formation of crypt abscesses, compromising the histoarchitecture. At greater magnification, changes suggestive of compromised epithelial integrity were noted, including flattening of enterocytes, epithelial ruptures and erosions (Figure 2H,I). In addition, hyperplasia, ulceration and a loss of intestinal crypt architecture were observed, with a marked depletion of goblet cells (Figure 2E). This depletion of goblet cells in the colon of mice with ulcerative colitis was further confirmed by immunohistochemical analysis of MUC‐2, as shown in Figure 5C,D. In addition, disorganisation of the mucosa was noted, characterised by irregular, branched crypts and partial or complete loss of crypts (Figure 2B,E,H). Although qualitatively, a noticeable improvement in the colonic wall morphology was observed in the HPE‐treated mice, suggesting attenuation of ulcerative colitis lesions (Figure 2C,F,I). The increased MUC‐2 expression shown in Figure 5C,D supports these findings.

### 3.3 Morphological Analysis of the Enteric Nervous System

**FIGURE 3:**
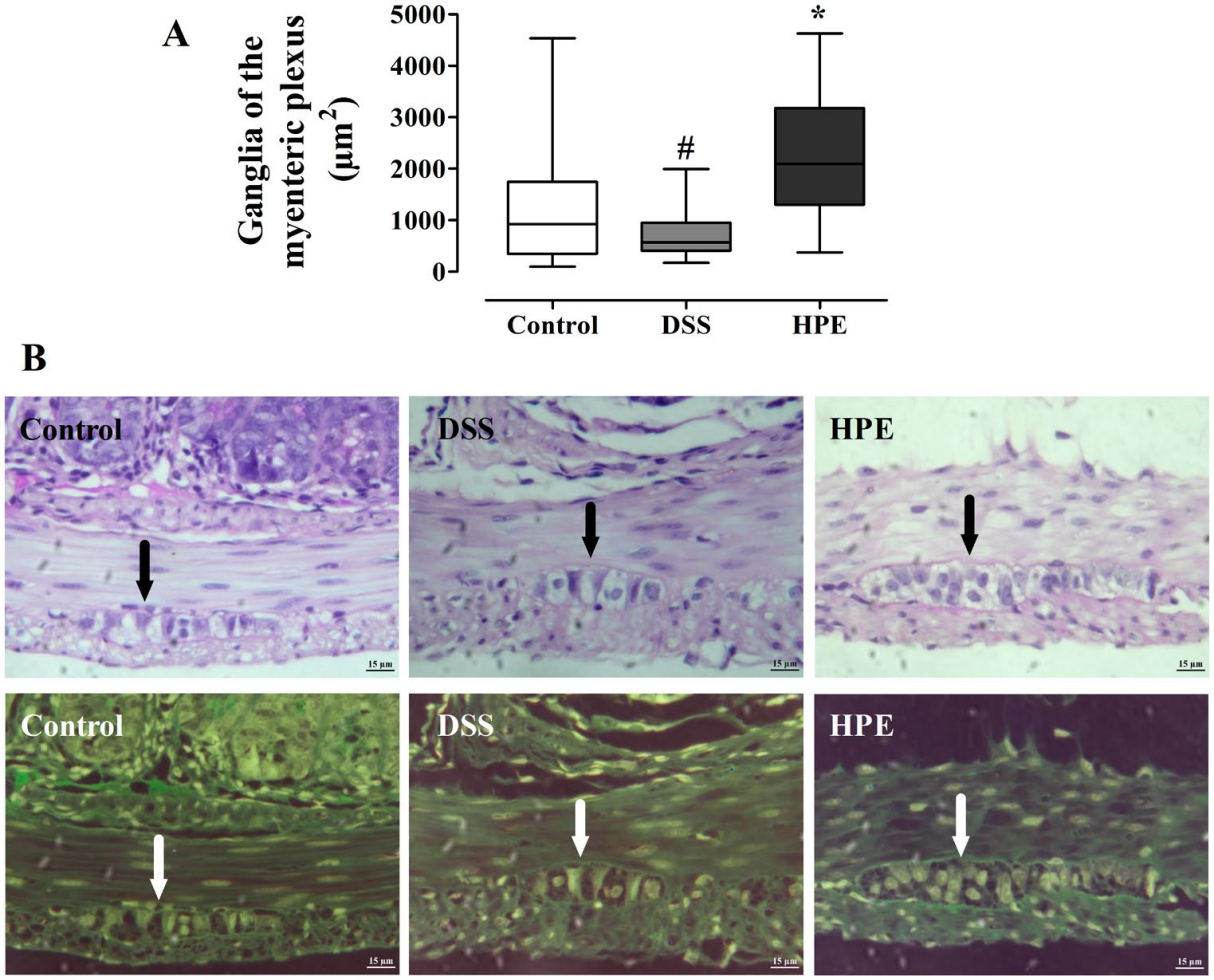
Histomorphometric analysis of the profile areas of the myenteric plexus in the colon of mice with DSS‐induced ulcerative colitis (A). Ulcerative colitis was induced by administration of 5% DSS in drinking water for 5 consecutive days. Mice were treated orally with vehicle (control and DSS group: Water, 1 mL/kg) or 100 mg/kg homogalacturonan from *P. edulis* (HPE group) once daily for 7 days. Results were presented as box‐and‐whisker plots with minimum and maximum values, medians and interquartile range and analyzed using the Kruskal–Wallis test followed by the Dunn post hoc test. # *p <* 0.05 compared with control group, *p < 0.05 compared with DSS group, *n* = 6. (B) Representative PAS‐stained photomicrographs of myenteric plexus ganglia (arrows) at 60× magnification from the muscular layer (inverted images shown for each group).

To evaluate the extent of damage caused by DSS‐induced ulcerative colitis and the therapeutic effects of HPE on the enteric nervous system of the colon of mice, we performed a histomorphometric analysis of the myenteric plexus ganglia. We found that DSS‐induced ulcerative colitis caused significant morphologic changes in the myenteric plexus ganglia. There was a significant reduction in the profile areas of the myenteric ganglia in the DSS group compared to the control group, with the median decreasing from 922.2 μm^2^ (350.4; 1748.1) in the control group to 572.2 μm^2^ (407.5; 953.5) in the DSS group (*p <* 0.05). On the other hand, HPE treatment led to a significant increase in the profile areas of the myenteric ganglion with a median of 2088.0 μm^2^ (1305.0; 3177.0) in the HPE group compared to the DSS group (*p <* 0.05; Figure 3).

### 3.4 Analysis of the Percentage of Collagen Type I and III per Area

**FIGURE 4:**
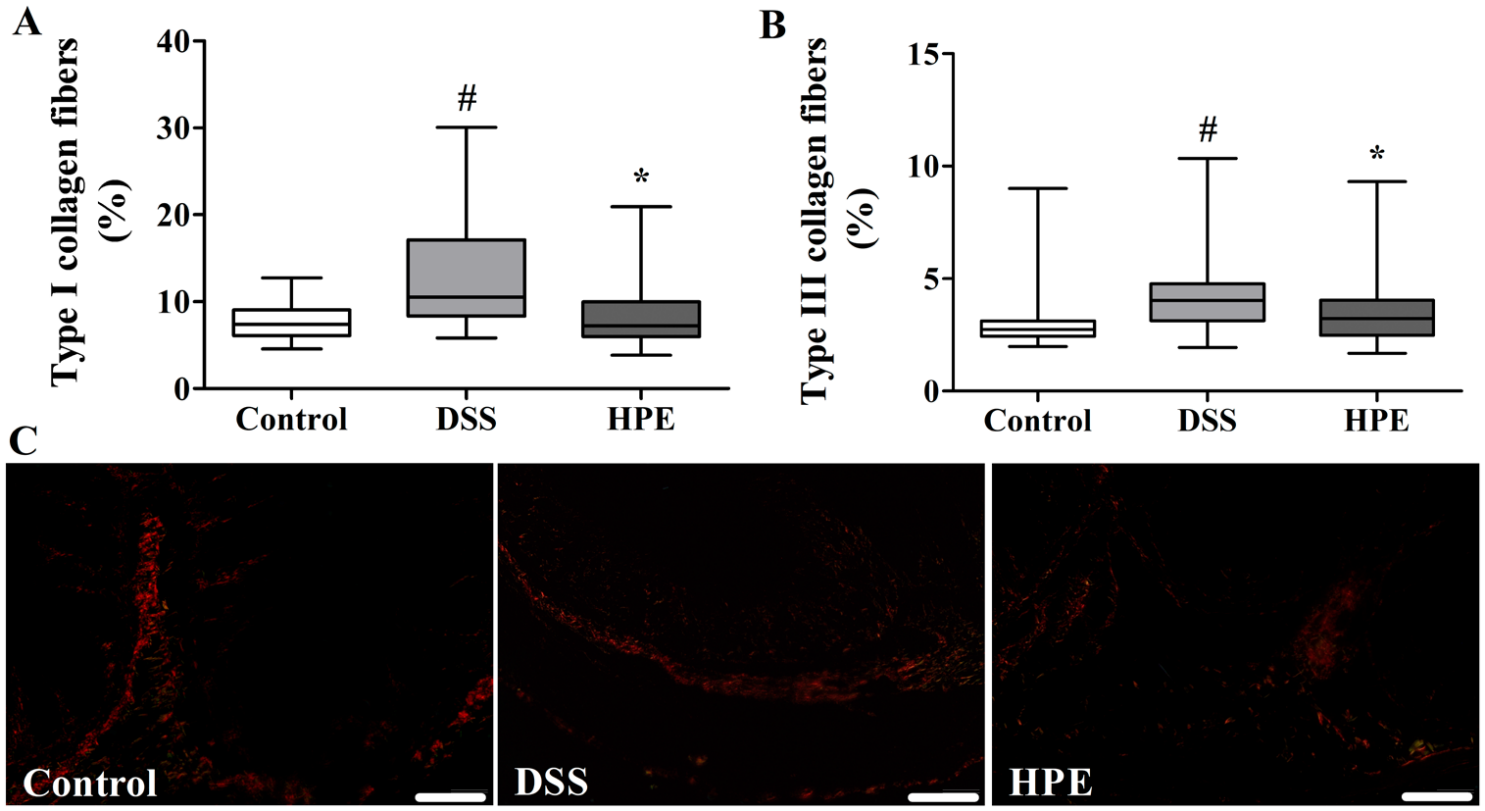
Dynamics of collagen remodelling in the extracellular matrix of the colon wall of mice with DSS‐induced ulcerative colitis treated with *P. edulis* homogalacturonan (HPE). (A) Type I collagen fibres (B) Type III collagen fibres. Ulcerative colitis was induced by administration of 5% DSS in drinking water for 5 consecutive days. Mice were treated orally with vehicle (control group and DSS: Water, 1 mL/kg) or 100 mg/kg HPE (HPE group) once daily for 7 days. Results were presented as box‐and‐whisker plots with minimum and maximum values, medians and interquartile range and analysed using the Kruskal–Wallis test followed by the Dunn post hoc test. # *p <* 0.05 compared with control group, *p < 0.05 compared with DSS group, *n* = 6. (C) Representative photomicrographs of the colon wall of mice showing type I (red and orange shading) and type III (green) collagen fibres labelled with Picro Sirius Red.

To investigate the dynamics of collagen remodelling in the extracellular matrix during DSS‐induced ulcerative colitis and the therapeutic effect of HPE in the colon wall of mice, we performed a quantitative analysis of type I and III collagen fibres. We found that both DSS‐induced ulcerative colitis and HPE treatment resulted in significant changes in collagen remodelling (Figure 4A–C).

DSS‐induced ulcerative colitis caused a significant increase in the deposition of type I collagen fibres in the colon wall of mice compared to the control group, with the median value increasing from 7.4 (6.1; 9.1) in the control group to 10.5 (8.3; 17.1) in the DSS group (*p <* 0.05). In contrast, HPE treatment resulted in a significant decrease in type I collagen fibre deposition, with a median value of 7.2 (5.9; 10.0) in the HPE group compared to the DSS group (*p <* 0.05; Figure 4A). Similarly, the deposition of type III collagen fibres in the colon wall of mice with DSS‐induced ulcerative colitis was also significantly increased compared to the control group, with the median value increasing from 2.7 μm (2.4; 3.1) in the control group to 4.0 μm (3.1; 4.7) in the DSS group (*p <* 0.05). HPE treatment significantly reduced the deposition of type III collagen, with a median of 3.2 μm (2.4; 4.1) in the HPE group compared to the DSS group (*p <* 0.05; Figure 4B).

### 3.5 Analysis of IEL, Mast Cell Distribution and Goblet Cells Expressing MUC‐2

**FIGURE 5:**
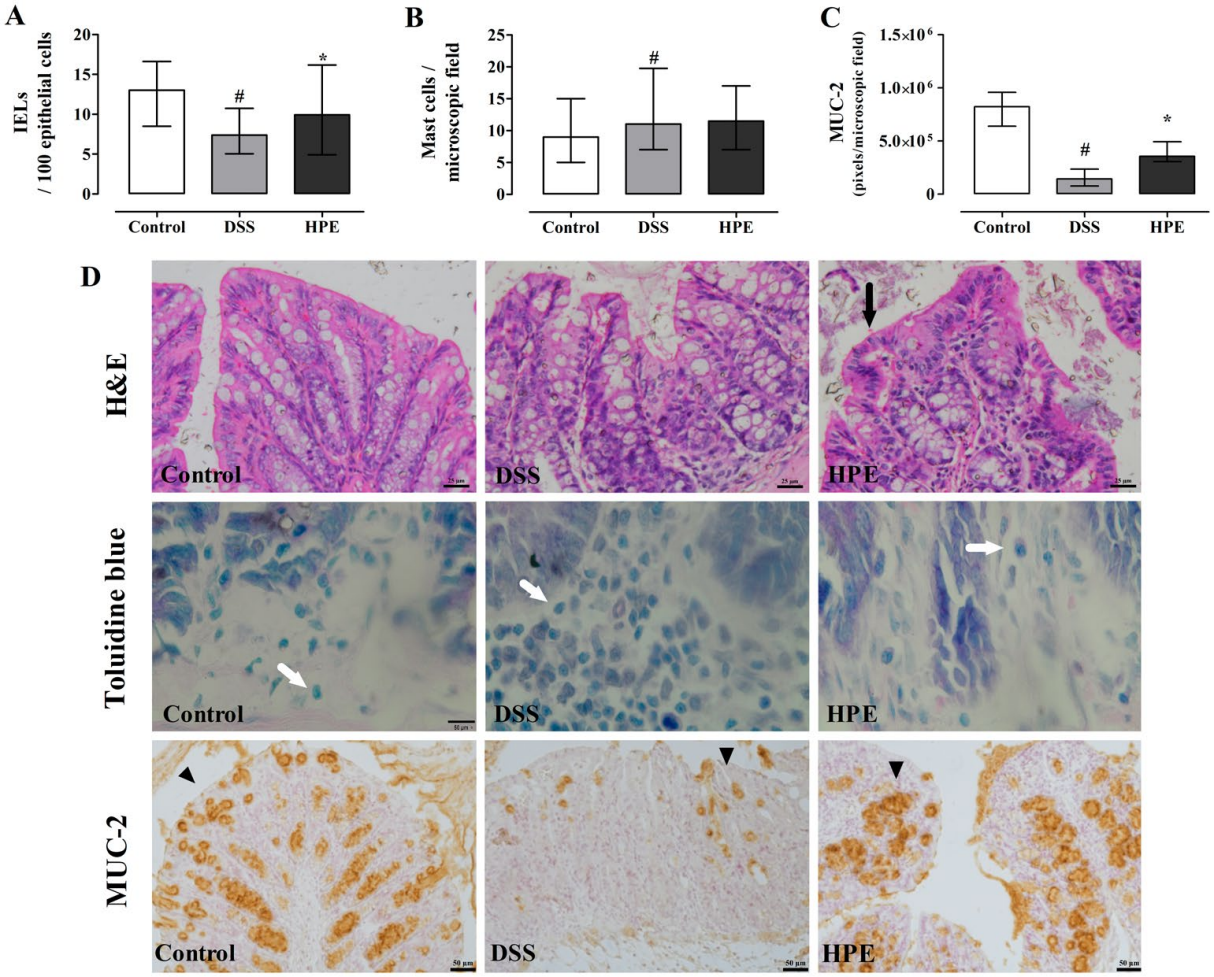
Distribution of intraepithelial lymphocytes (IELs) and mast cells in the colonic mucosa of mice with DSS‐induced ulcerative colitis treated with homogalacturonan from *P. edulis* (HPE). (A) Number of IELs per 100 epithelial cells. (B) Number of mast cells per microscopic field. Ulcerative colitis was induced by administration of 5% DSS in drinking water for 5 consecutive days. Mice were treated orally with vehicle (control and DSS group: Water, 1 mL/kg) or 100 mg/kg HPE (HPE group) once daily for 7 days. Results were expressed as median with interquartile range and analysed using the Kruskal–Wallis test followed by the Dunn post hoc test. # *p <* 0.05 compared with control group, *p < 0.05 compared with DSS group, *n* = 6. (D) Representative photomicrographs of colonic mucosa from mice with HE‐stained IELs (arrows), toluidine blue‐stained mast cells (white arrows) and MUC‐2 expression in goblet cells (arrowheads).

To investigate the distribution of IELs, mast cells and goblet cells expressing MUC‐2 in mice with DSS‐induced ulcerative colitis, and the therapeutic effect of homogalacturonan (HPE) extracted from *P. edulis* on the colonic mucosa, a detailed quantitative analysis was performed. DSS‐induced ulcerative colitis markedly altered the distribution and expression of these cells, while treatment with HPE partially restored the integrity of the intestinal mucosa (Figure 5).

Quantitative analysis showed that DSS‐induced ulcerative colitis significantly reduced the number of IELs per 100 epithelial cells compared to the control group, with the median value decreasing from 13.0 (8.4; 16.6) in the control group to 7.3 (5.1; 10.75) in the DSS group (*p* < 0.05). In contrast, HPE treatment increased the number of IELs, reaching a median of 9.9 (4.9; 16.1) per 100 epithelial cells compared to the DSS group (*p* < 0.05; Figure 5A).

DSS‐induced ulcerative colitis significantly increased the number of mast cells per microscopic field compared to the control group, with the median value increasing from 9 (5; 15) in the control group to 11 (6.2; 19) in the DSS group (*p* < 0.05). In contrast, HPE treatment did not significantly change mast cell numbers, presenting a median of 11.5 (7; 17) in the HPE group compared to the DSS group (*p* > 0.05; Figure 5B).

DSS‐induced ulcerative colitis significantly reduced MUC‐2 expression in the colonic goblet cells of the colon compared to the control group (*p* < 0.05). In contrast, HPE treatment significantly increased MUC‐2 expression compared to the DSS group (*p* < 0.05; Figure 5C). Representative photomicrographs highlighting MUC‐2‐positive goblet cells are shown in Figure 5D.

### 3.6 Pharmacokinetic and Toxicological Predictions

The results in Table 3 show the absorption properties of galacturonic acid [33]. In terms of water solubility, HPE reached a low value of −1.9 mol L^−1^. The Caco‐2 cell permeability model (lineage of heterogeneous cells of colorectal human epithelial adenocarcinoma) was used as a parameter to predict the oral absorption of HPE. The value obtained was considered low (−0.217 × 10^−6^ cm s^−1^). One of the most important parameters for new compounds is the analysis of absorption in the human intestine (AIH). HPE showed an absorption of 8.071%, which means that it is not absorbed through the gastrointestinal tract. HPE cannot be absorbed through human skin (−2.735 cm h^−1^) and does not bind to or inhibit the substrate of P‐glycoprotein [34, 46].

**TABLE 3:** Absorption characteristics of galacturonic acid.

Model name	Predicted value
Water solubility (log mol/L)	−1.9
Caco_2_ permeability (log Papp in 10^−6^ cm/s)	−0.217
Intestinal absorption (human) (% Absorbed)	8.071
Skin permeability	−2.735
P‐glycoprotein substrate	No
P‐glycoprotein I inhibitor	No

The distribution properties of galacturonic acid are shown in Table 4. In terms of volume distribution at steady state (VDss), HPE showed a reduced value (−0.471 L kg^−1^). The unbound fraction of the compound was measured by human blood (Fu), 0.779 [47]. The result of the blood–brain barrier (BBB) penetration potential log BB −0.68 showed that HPE has a low potential to cross the blood–brain barrier, with a value below 0.1 [48]. As for penetration into the central nervous system (CNS), HPE showed a result of log PS −4.028, indicating low penetration into the CNS, as it is below the value of log PS −3.0.

**TABLE 4:** Distribution characteristics of galacturonic acid.

Model name	Predicted value
VDss (human) (log L/kg)	−0.471
Fraction unbound (human) (Fu)	0.779
BBB permeability (log BB)	−0.69
CNS permeability (log PS)	−4.028

The results of the metabolic properties of galacturonic acid are presented in Table 5, which shows the interaction of HPE with cytochrome P450 protein (CYP) families. The main enzymes involved in metabolism were investigated, including CYP2D6, CYP3A4, CYP1A2, CYP2C19 and CYP2C9. HPE does not bind to the substrate and does not inhibit CYP2D6 and CYP3A4. It also did not inhibit CYP1A2, CYP2C19 and CYP2C9 [49].

**TABLE 5:** Metabolic characteristics of galacturonic acid.

Model name	Predicted value
CYP2D6 substrate	No
CYP3A4 substrate	No
CYP1A2 inhibitor	No
CYP2C19 inhibitor	No
CYP2C9 inhibitor	No
CYP2D6 inhibitor	No
CYP3A4 inhibitor	No

The excretion characteristics of the galacturonic acid results are shown in Table 6. The HPE result for total clearance was 0.662 mL min kg ^−1^. HPE does not bind to the renal OCT2 substrate [50]. Table 7 shows the results of the toxicological properties of galacturonic acid (Table 7). HPE showed no prediction for the AMES test (bacterial reverse mutation test); it showed a high value (1.838 mg kg day^−1^) for the maximum tolerated dose (human) (reference value: high > 0.477 mg kg day^−1^). HPE was not able to inhibit the human ether‐a‐go‐go gene (hERG) I or II.

**TABLE 6:** Excretion characteristics of galacturonic acid.

Model name	Predicted value
Total clearance (log mL/min/kg)	0.662
Renal OCT2 substrate	No

**TABLE 7:** Toxicological properties of galacturonic acid.

Model name	Predicted value
AMES toxicity	No
Max. tolerated dose (human) (log mg/kg/day)	1.838
hERG I inhibitor	No
hERG II inhibitor	No
Oral rat acute toxicity (LD50)	1.314
Oral rat chronic toxicity (LOAEL) (log mg/kg_bw/day)	4.037
Hepatotoxicity	No
Skin sensitisation	No
*T. pyriformis* toxicity	0.285
Minnow toxicity (log mM)	4.57

According to Archer, 1985, HPE had a low oral rat acute toxicity (LD50—lethal dose, 50%): 1314 mg kg^−1^ [51]. For oral chronic toxicity in rats (LOAEL), HPE showed a result of log 4037 mg kg_bw day^−1^. In addition, HPE showed no hepatotoxicity or skin sensitization. The HPE result for the toxicity of *T. pyriformis* was 0.285 mg kg^−1^ and thus non‐toxic (reference value > 0.5 mg kg^−1^). The HPE showed a low toxicity of Minnow (4.57 mM). According to the reference, LD50 values below 0.5 mM are considered high acute toxicity [52].

### 3.7 Molecular Docking

The results of the molecular docking of the monosaccharide α‐D‐Gal*p*A with 1XAW (occludin), 5Y2T (PPAR‐γ receptor), 6BSC (mucin 1) and 6TM6 (mucin) are shown in Table 8.

**TABLE 8:** Molecular affinity parameters of the compound α‐D‐Gal*p*A with 1XAW, 5Y2T, 6BSC and 6TM6.

Complex (ligand‐protein)	∆G_bind_ (kcal mol^−1^)	Ki (μM)	Amino acids that interact through hydrogen bonds	Amino acids that make hydrophobic interactions
α‐D‐Gal*p*A/6bsc	−5.09	184.47 μM	ARG1095, LEU1045, LYS1093 and PHE1047	HIS1048, PHE1044, PHE1094 and SER1046
α‐D‐Gal*p*A/1xaw	−5.01	213.78 μM	LYS497, LYS501, LYS504 and TYR443	CYS500, GLN447 and LEU450
α‐D‐Gal*p*A/5y2t	−3.46	2.91 μM	HIS323, HIS449, TYR327 and TYR473	CYS285, GLN286, ILE326, LEU453, LEU465, LEU469, LYS367, PHE282, PHE363 and SER289
α‐D‐Gal*p*A/6tm6	−3.22	4.33 mM	ARG1339, CYS1338, CYS1357, GLU1349, ILE1336 and LEU1348	ASP1358, GLU1337 and SER1347

*Note:* The mucin 1 (6bsc) and the ligand α‐D‐Gal*p*A showed the highest molecular affinity, with a binding energy of −5.09 kcal mol^−1^ and an inhibition constant of 184.47 μM. The α‐D‐Gal*p*A/6BSC complex formed four hydrogen bonds (ARG1095, LEU1045, LYS1093 and PHE1047) at the active site of the protein and four interactions through hydrophobic bonds (Figure 6; Table 8).

**FIGURE 6:**
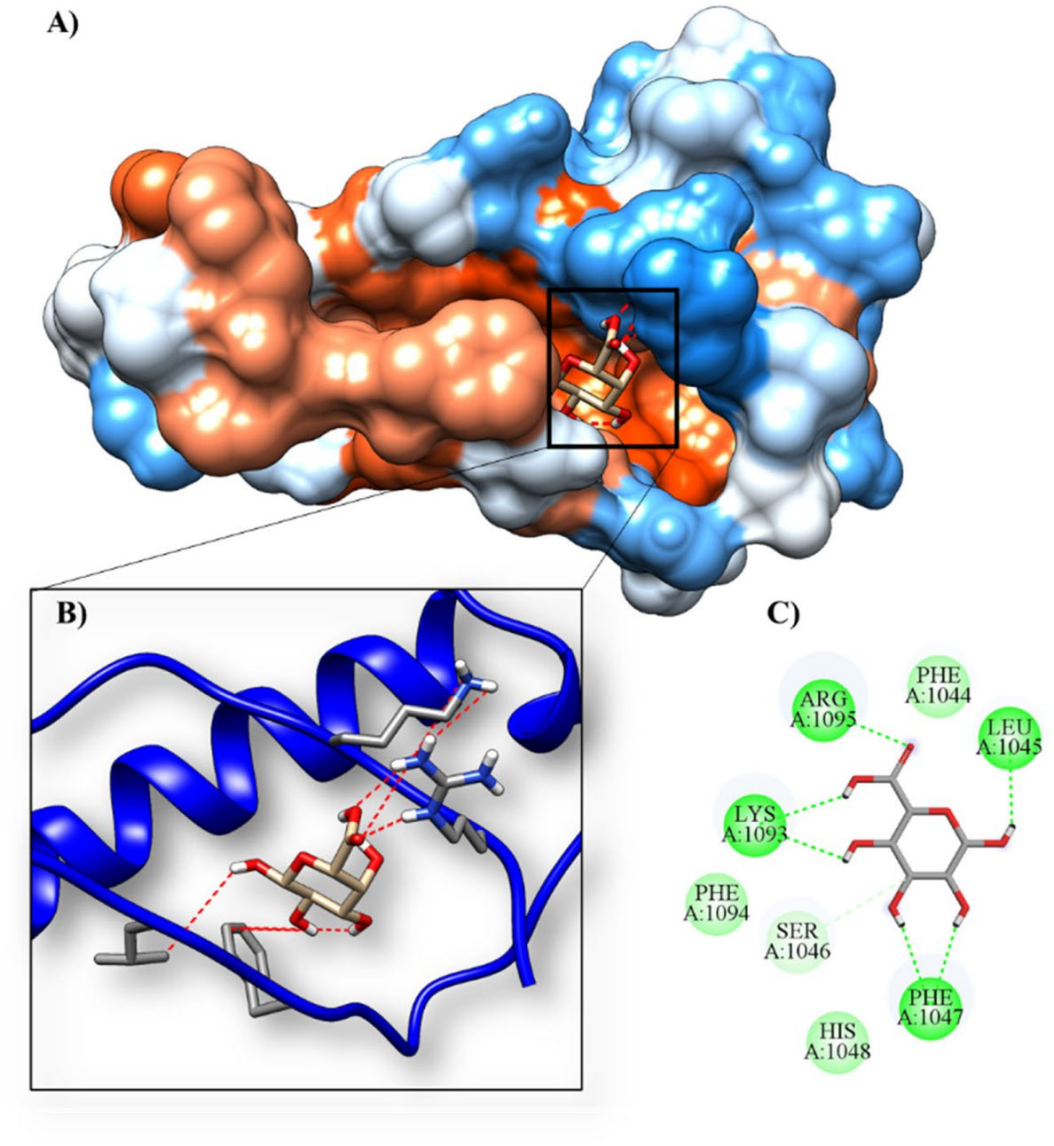
Molecular docking of the protein–ligand complex with the 6BSC protein and the α‐D‐GalpA ligand. (A) Three‐dimensional structure of the MUC1 SEA domain in complex with the ligand, highlighting its spatial arrangement. (B) Active site of the protein, indicating the specific region where the ligand was docked. (C) Two‐dimensional representation of the molecular interactions established between the ligand and the binding site residues, including hydrogen bonds and hydrophobic interactions.

**FIGURE 7:**
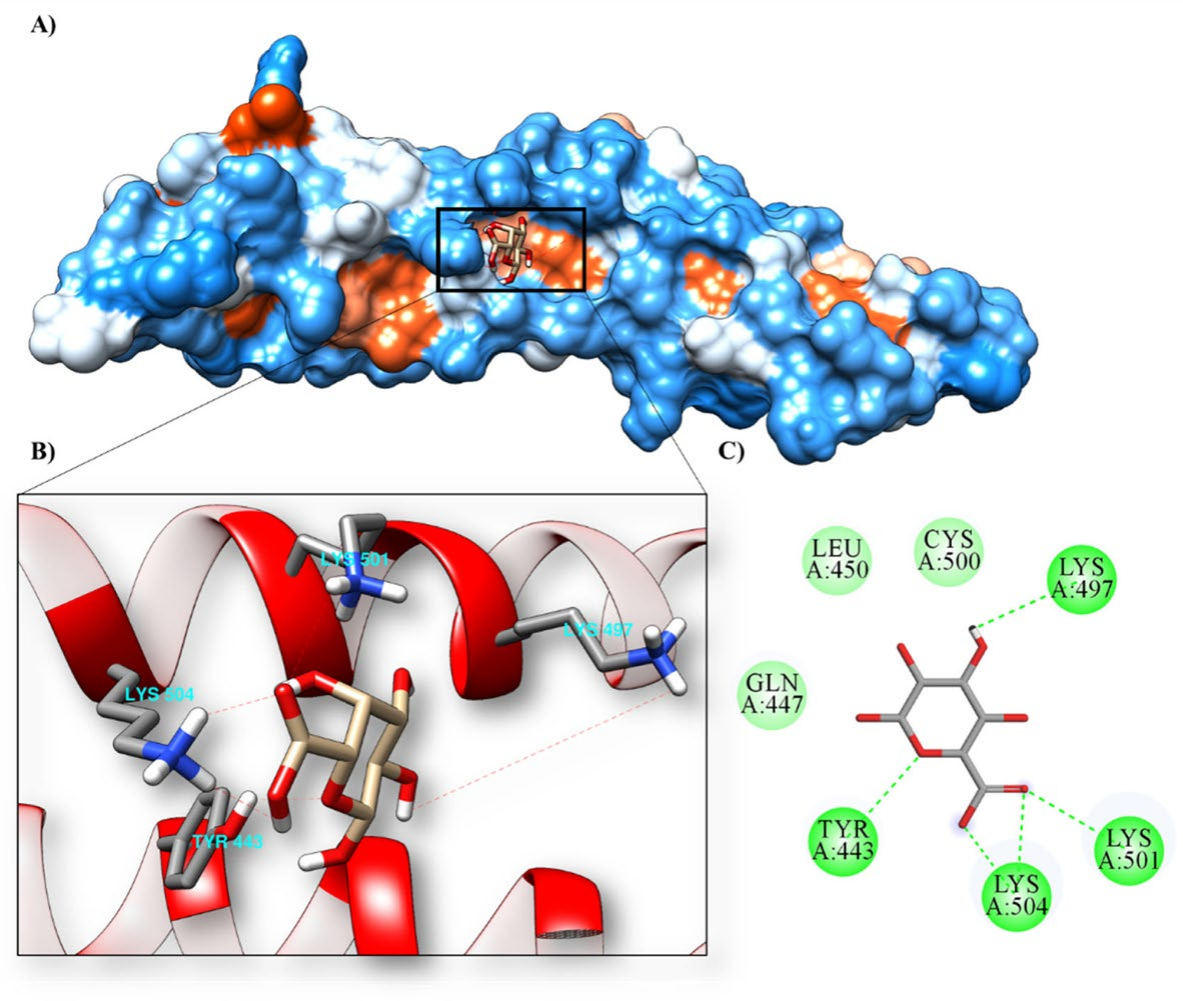
Molecular docking of the protein‐ligand complex with the 1XAW (occludin) protein and the α‐D‐Gal*p*A ligand, (A) contact surface, (B) hydrogen bonds at the active site, (C) 2D interactions.

The transmembrane protein 1XAW (occludin) and the ligand α‐D‐Gal*p*A showed a molecular affinity with a binding energy of −5.01 kcal mol^−1^ and an inhibition constant of 213.78 μM. The α‐D‐Gal*p*A/1XAW complex also formed four hydrogen bonds (LYS497, LYS501, LYS504 and TYR443) at the active site of the protein and three interactions through hydrophobic bonds (CYS500, GLN447 and LEU450) (Figure 7; Table 8).

**FIGURE 8:**
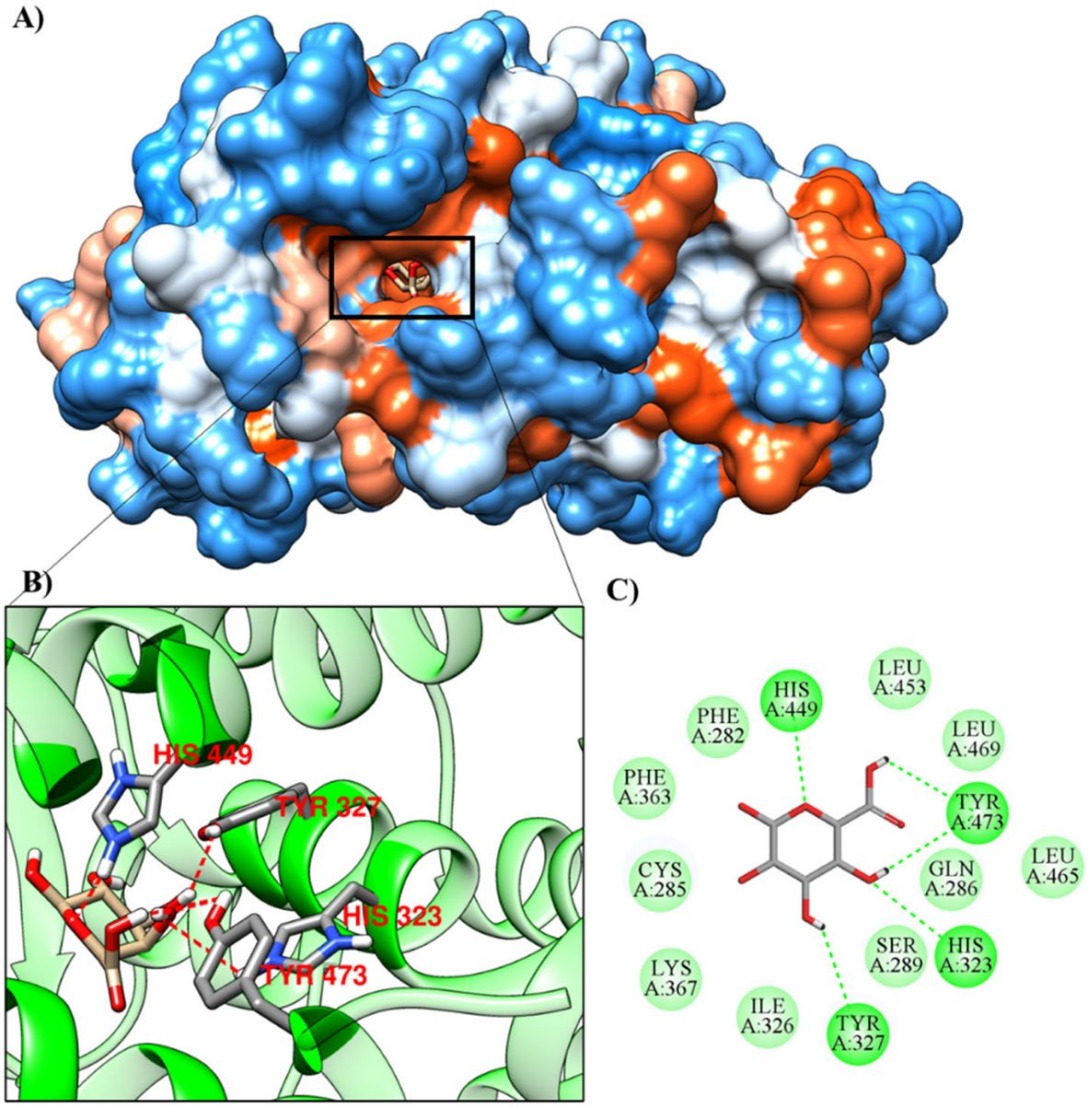
Molecular docking of the protein‐ligand complex with the 5Y2T protein and the α‐D‐Gal*p*A ligand, (A) contact surface, (B) hydrogen bonds at the active site, (C) 2D interactions.

The PPAR‐γ receptor (5Y2T) with the monosaccharide α‐D‐Gal*p*A showed a binding affinity of −3.46 kcal·mol^−1^ and an inhibition constant of 2.91 μM, and the results demonstrate that the ligand interacts with the amino acids HIS323, HIS449, TYR327 and TYR473 via hydrogen bonds and with CYS285, GLN286, ILE326, LEU453, LEU465, LEU469, LYS367, PHE282, PHE363 and SER289 by hydrophobic bonds (Figure 8; Table 8).

**FIGURE 9:**
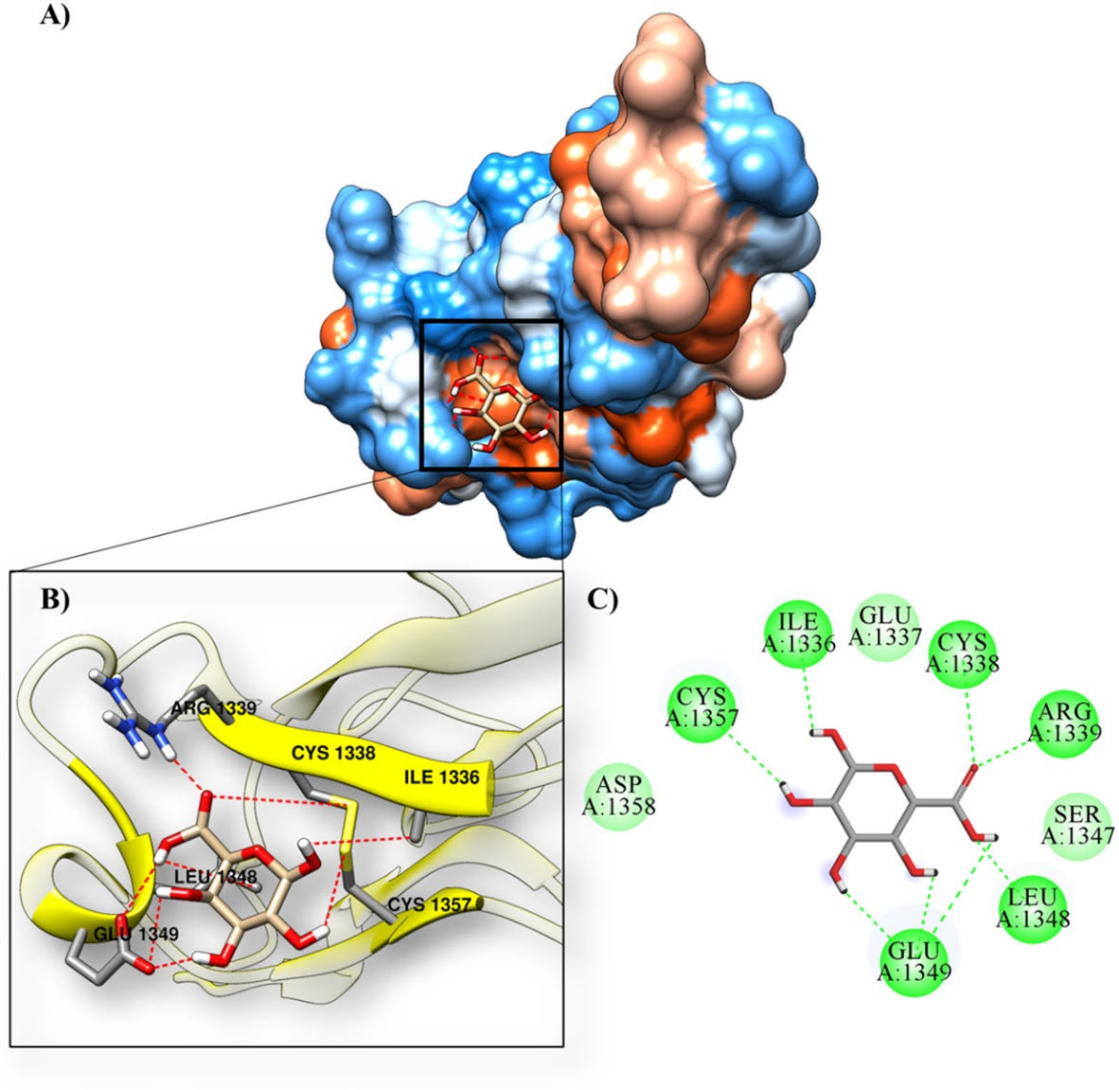
Molecular docking of the protein‐ligand complex with the 6TM6 protein and the α‐D‐Gal*p*A ligand, (A) contact surface, (B) hydrogen bonds at the active site, (C) 2D interactions.

The mucin (6TM6) with the monosaccharide α‐D‐Gal*p*A showed a binding affinity of −3.22 kcal·mol^−1^ and an inhibition constant of 4.33 mM, and the results show that the ligand interacts with the amino acids ARG1339, CYS1338, CYS1357, GLU1349, ILE1336 and LEU1348 via hydrogen bonds and ASP1358, GLU1337 and SER1347 via hydrophobic bonds (Figure 9; Table 8).

## 4 Discussion

In the present study, we demonstrated that DSS‐induced ulcerative colitis causes significant morphologic changes in the colonic wall, including changes in the thickness of the muscular, submucosal and mucosal layers, as well as changes in the depth and width of the intestinal crypts, collagen accumulation, morphological changes in the ganglia of the myenteric plexus, and changes in the distribution of mast cells and intraepithelial lymphocytes (IELs), as well as a marked reduction in MUC‐2 expression in goblet cells. In addition, qualitative histopathologic analysis revealed several changes in the colonic wall, including the presence of intense and diffuse inflammatory infiltrates in the lamina propria and submucosa. Epithelial flattening, ruptures, erosions and ulcerations of the mucosa, depletion of goblet cells, distortion and loss of intestinal crypts, and the formation of crypt abscesses were also observed.

The experimental protocol used in this study, which includes the induction of ulcerative colitis with DSS, a widely recognised model [53], ensures that the observed harms result directly from the chosen experimental model. Moreover, the effects observed after treatment with *P. edulis* homogalacturonan (HPE), properly obtained, purified, characterised and administered by gavage, guarantee the reproducibility of our results. Thus, we were able to demonstrate that treatment with HPE was effective and mitigated the damage caused, thereby promoting tissue recovery and enhancing the therapeutic potential of natural products in the treatment of ulcerative colitis.

Previous studies have shown the importance of maintaining the structural integrity of the colonic wall and have demonstrated the benefits of plant‐derived polysaccharides in achieving this objective [8, 9, 10, 11, 54, 55]. Accordingly, the present study focuses on evaluating the effects of HPE on alleviating the damage associated with ulcerative colitis.

As mentioned above, HPE treatment improved all assessed morphological parameters compared to the untreated group and also restored the expression of MUC‐2 in the goblet cells, which was significantly reduced in DSS‐induced colitis. However, the mechanisms involved in the restoration of morphological patterns cannot be fully elucidated on the basis of this study alone. It can be concluded that HPE treatment restored the thickness patterns of the muscle, submucosa and mucosa layers, as well as the depth and width of the intestinal crypts and the height and width of the enterocytes, making them more similar to those observed in the control group.

The restoration of MUC‐2 expression suggests a potential protective effect on goblet cells, which are essential for maintaining the mucus layer and the integrity of the epithelial barrier. It has been demonstrated that treatment with polysaccharides extracted from jambu [13], guavira [9], tamarillo [11] and DIREN, an ethnic medicine from SHE [56], attenuated morphological damage and protected goblet cells in experimental models of ulcerative colitis. In addition, polysaccharides from the peels of yellow passion fruit have been shown to reduce pro‐inflammatory cytokines, preserve the mucus barrier, increase anti‐inflammatory cytokines and restore antioxidant levels, thereby promoting inflammatory homeostasis [8, 11].

In some cases where an increase in the thickness of certain morphological parameters has been observed, it is important to consider that the migration of immune system cells may have contributed to this thickening. An example of this is the submucosa, which showed a thickening accompanied by an intense inflammatory infiltrate, as shown by histopathological analysis. Following this reasoning and taking into account the inflammation induced by colitis and the recruitment of immune cells, the thickening of parameters such as the mucosa may be associated with the presence of edema and the formation of abscesses [57].

Several authors have reported morphologic changes in the colonic wall in experimental models of DSS‐induced colitis, including atrophy of the muscular layer and loss of intestinal motility, reduced length and changes in the histoarchitecture of the colonic mucosa and epithelium [8, 9, 13, 16, 56].

DSS‐induced ulcerative colitis led to a flattening of the enterocytes, characterised by a reduction in height and an increase in width. This change indicates a loss of epithelial barrier integrity, as the distance between the intestinal lumen and the lamina propria was reduced. This finding was also confirmed in the qualitative histopathologic analysis. The decreased distance associated with the reduction in goblet cells, as demonstrated in the histopathologic analysis and further confirmed by the immunohistochemical analysis of MUC‐2, may facilitate the translocation of microorganisms from the lumen, increasing the risk of inflammation and intestinal dysfunction. The decrease in goblet cells was associated with lower expression of MUC‐2, which impairs the protective mucosal barrier and may exacerbate mucosal injury. The loss of epithelial integrity increased the susceptibility of the mucosa to damage and facilitated bacterial translocation, thereby exacerbating the inflammatory process and disrupting intestinal homeostasis [3, 4, 58]. In this study, treatment with HPE showed a protective effect and promoted partial restoration of enterocyte height and reduction in width, which enhances the restoration of the mucus layer and protection of the epithelium.

An important morphological parameter analysed in this study was the area of the enteric nervous system (ENS) ganglia, which is essential for intestinal motility and inflammation regulation [59]. Recent studies have shown that ulcerative colitis models exhibit significant changes in the ENS that negatively affect intestinal motility and lead to motor dysfunction such as diarrhoea [15, 16]. In our study, we observed that the reduction in the area of myenteric ganglia induced by ulcerative colitis was reversed by treatment with HPE. Interestingly, the ganglia area in the treated group not only recovered but also exceeded the average values of the healthy control group. This finding suggests a possible neuroprotective effect of HPE, possibly stimulating neuronal synthesis and contributing to tissue repair. However, this hypothesis still requires further investigation to be confirmed. It is noteworthy that these results contrast with those of Braga et al. [11], who showed that treatment with DSS resulted in a significant increase in the profile area of myenteric ganglia compared to controls, while the administration of polysaccharides from tamarillo pulp effectively reduced this parameter. These divergent results may be due to differences in the structural composition and biological activity of the polysaccharides used, as well as variations in the experimental models, treatment regimens, or the extent of extracellular matrix remodelling, which was also observed in our study.

In ulcerative colitis, there is a significant increase in the deposition of type I and type III collagen, which leads to fibrosis of the intestinal wall, impaired motility and increased tissue rigidity [16, 23, 24]. The altered dynamics of collagen fibre remodelling are also associated with increased peripheral sensitization of sensory neurons, which contributes to the visceral pain characteristic of ulcerative colitis [17]. In this study, a significant increase in type I and III collagen deposition was observed in DSS‐induced ulcerative colitis in mice. However, it remains unclear whether this represents the early stages of fibrosis development. Treatment with HPE reduced the deposition of type I and type III collagen, indicating a possible antifibrotic effect that needs to be further investigated. Recent studies have shown that polysaccharides can modulate the deposition of collagen fibres, attenuate colonic fibrosis and reduce inflammation [9, 13, 56].

In the present study, qualitative analysis showed the presence of an intense inflammatory infiltrate consisting predominantly of neutrophils and lymphocytes. Quantitative analysis showed a reduction in the number of IELs and an increase in mast cells in untreated mice that had colitis. Treatment with HPE, on the other hand, led to a significant increase in the proportion of IELs without affecting the distribution of mast cells. The observed reduction in IELs is directly related to damage to the colonic epithelium, as evidenced by the presence of erosions on the epithelial surface. The loss of the epithelial surface leads to a reduction in epithelial cells and thus a decrease in IELs. As the treatment mitigated the damage, the restoration of epithelial integrity allowed the migration of these immune cells, which play a crucial role in the inflammatory response associated with ulcerative colitis. IELs and mast cells play a central role in the immunopathology of ulcerative colitis [18] and the observed restoration of MUC‐2 expression suggests that HPE also contributed to the restoration of goblet cell secretory barrier function, which is essential for mucosal homeostasis [9, 11].

To further verify the mechanism of HPE, we performed a pharmacokinetic analysis and investigated the possible molecular mechanism by in silico analysis. HPE shows low absorbability through the gastrointestinal tract or human skin and does not bind or inhibit the substrate of P‐glycoprotein. In addition, it does not cross the blood–brain barrier and CNS and does not inhibit cytochrome P450 protein families, suggesting that it is non‐toxic. Importantly, HPE has a high maximum tolerated dose value in humans and has low acute oral toxicity in rats, no hepatotoxicity, or skin sensitization. The ligand α‐D‐Gal*p*A showed molecular affinity to occludin (1XAW), PPAR‐γ receptor enzyme (5Y2T) and mucin (6TM6), with the highest molecular affinity to mucin 1 (6BSC). The literature indicates that those polysaccharides from natural sources have intricate structural complexity and diverse biological activities [60]. This complexity suggests that their bioactive effects may result from dynamic interactions with a variety of proteins. In this context, we propose that the beneficial effects of HPE may be mediated, at least in part, by its interaction with the identified proteins. However, elucidation of the precise molecular mechanisms underlying these interactions requires further in‐depth studies.

In conclusion, our results show that HPE treatment was effective in attenuating most of the damage caused by DSS‐induced ulcerative colitis, as evidenced by the improvements in the morphological parameters assessed and the restoration of MUC‐2 expression in goblet cells. These results support the potential of polysaccharides as a promising approach for the treatment of ulcerative colitis, with the potential to reduce associated complications and enhance the therapeutic use of natural products. Despite the limitations of this study, such as the fixed HPE dosage and the lack of detailed analysis of the underlying mechanisms, it provides valuable morphological evidence for the effect of plant‐derived polysaccharides and emphasises the importance of exploring safe and effective natural therapies for ulcerative colitis.

## Author Contributions

**Samilla Santos Souza Mazeti:** data curation (supporting), formal analysis (equal), investigation (equal), methodology (lead), project administration (equal), writing – original draft (lead). **Ariane Aviles Turini:** data curation (supporting), methodology (supporting). **Laryssa Regis Bueno:** conceptualization (supporting), data curation (supporting), writing – original draft (supporting). **Cleiane Dias Lima:** formal analysis (supporting), methodology (supporting), software (supporting). **Ruan Sousa Bastos:** formal analysis (supporting), methodology (supporting), software (supporting). **Jefferson Almeida Rocha:** methodology (equal), software (equal), writing – review and editing (equal). **Lucimara Mach Côrtes Cordeiro:** data curation (supporting), funding acquisition (equal), investigation (equal), project administration (supporting), supervision (supporting), writing – review and editing (supporting). **Daniele Maria‐Ferreira:** conceptualization (equal), funding acquisition (supporting), project administration (equal), validation (equal), writing – review and editing (equal). **Marcelo Biondaro Gois:** conceptualization (lead), funding acquisition (supporting), investigation (equal), methodology (equal), project administration (lead), resources (equal), supervision (supporting), validation (lead), writing – review and editing (lead).

## Conflicts of Interest

The authors declare no conflicts of interest.

## Data Availability

Data available on request from the authors.

## References

[1] R. Ungaro , S. Mehandru , P. B. Allen , L. Peyrin‐Biroulet , and J.‐F. Colombel , “Ulcerative Colitis,” Lancet 389 (2017): 1756–1770, 10.1016/S0140-6736(16)32126-2. 27914657 PMC6487890

[2] J. D. Feuerstein and A. S. Cheifetz , “Ulcerative Colitis: Epidemiology, Diagnosis, and Management,” Mayo Clinic Proceedings 89 (2014): 1553–1563, 10.1016/j.mayocp.2014.07.002. 25199861

[3] A. Saez , B. Herrero‐Fernandez , R. Gomez‐Bris , H. Sánchez‐Martinez , and J. M. Gonzalez‐Granado , “Pathophysiology of Inflammatory Bowel Disease: Innate Immune System,” International Journal of Molecular Sciences 24 (2023): 1526, 10.3390/ijms24021526. 36675038 PMC9863490

[4] G. Świrkosz , A. Szczygieł , K. Logoń , M. Wrześniewska , and K. Gomułka , “The Role of the Microbiome in the Pathogenesis and Treatment of Ulcerative Colitis—A Literature Review,” Biomedicine 11 (2023): 3144, 10.3390/biomedicines11123144. PMC10740415 38137365

[5] M. Imbrizi , F. Magro , and C. S. R. Coy , “Pharmacological Therapy in Inflammatory Bowel Diseases: A Narrative Review of the Past 90 Years,” Pharmaceuticals (Basel) 16 (2023): 1272, 10.3390/ph16091272. 37765080 PMC10537095

[6] Y. Dilixiati , A. Aipire , M. Song , et al., “The Potential Role of Plant Polysaccharides in Treatment of Ulcerative Colitis,” Pharmaceutics 16 (2024): 1073, 10.3390/pharmaceutics16081073. 39204418 PMC11360206

[7] W. Chen , H. Fan , R. Liang , R. Zhang , J. Zhang , and J. Zhu , “ *Taraxacum officinale* Extract Ameliorates Dextran Sodium Sulphate‐Induced Colitis by Regulating Fatty Acid Degradation and Microbial Dysbiosis,” Journal of Cellular and Molecular Medicine 23 (2019): 8161–8172, 10.1111/jcmm.14686. 31565850 PMC6850927

[8] L. R. Bueno , B. da Silva Soley , K. Y. Abboud , et al., “Protective Effect of Dietary Polysaccharides From Yellow Passion Fruit Peel on DSS‐Induced Colitis in Mice,” Oxidative Medicine and Cellular Longevity 2022 (2022): 6298662, 10.1155/2022/6298662. 36285298 PMC9588357

[9] N. de Oliveira , V. S. Schneider , L. R. Bueno , et al., “CPW Partially Attenuates DSS‐Induced Ulcerative Colitis in Mice,” Food Research International 173 (2023): 113334, 10.1016/j.foodres.2023.113334. 37803644

[10] C. S. Schiebel , L. R. Bueno , R. B. Pargas , et al., “Exploring the Biological Activities and Potential Therapeutic Applications of Agro‐Industrial Waste Products Through Non‐Clinical Studies: A Systematic Review,” Science of the Total Environment 950 (2024): 175317, 10.1016/j.scitotenv.2024.175317. 39111448

[11] L. L. V. d. M. Braga , C. S. Schiebel , G. Simão , et al., “Type I Arabinogalactan and Methyl‐Esterified Homogalacturonan Polysaccharides From Tamarillo (*Solanum betaceum* cav.) Fruit Pulp Ameliorate DSS‐Induced Ulcerative Colitis,” Pharmaceuticals (Basel) 18 (2025): 461, 10.3390/ph18040461. 40283898 PMC12030512

[12] M. A. Odenwald and J. R. Turner , “The Intestinal Epithelial Barrier: A Therapeutic Target?,” Nature Reviews. Gastroenterology & Hepatology 14 (2017): 9–21, 10.1038/nrgastro.2016.169. 27848962 PMC5554468

[13] D. Maria‐Ferreira , A. M. Nascimento , T. R. Cipriani , et al., “Rhamnogalacturonan, a Chemically‐Defined Polysaccharide, Improves Intestinal Barrier Function in DSS‐Induced Colitis in Mice and Human Caco‐2 Cells,” Scientific Reports 8 (2018): 12261, 10.1038/s41598-018-30526-2. 30115942 PMC6095889

[14] R. J. Porter , R. Kalla , and G.‐T. Ho , “Ulcerative Colitis: Recent Advances in the Understanding of Disease Pathogenesis,” F1000Research 9 (2020): 1–13. 10.12688/f1000research.20805.1. PMC7194476 32399194

[15] H. I. R. Magalhães and P. Castelucci , “Enteric Nervous System and Inflammatory Bowel Diseases: Correlated Impacts and Therapeutic Approaches Through the P2X7 Receptor,” World Journal of Gastroenterology 27 (2021): 7909–7924, 10.3748/wjg.v27.i46.7909. 35046620 PMC8678817

[16] P. d. S. Watanabe , A. M. Cavichioli , J. D'Arc de Lima Mendes , et al., “Colonic Motility Adjustments in Acute and Chronic DSS‐Induced Colitis,” Life Sciences 321 (2023): 121642, 10.1016/j.lfs.2023.121642. 36990176

[17] M. D. V. da Silva , L. da Silva Bonassa , M. Piva , et al., “Perineuronal Net in the Extrinsic Innervation of the Distal Colon of Mice and Its Remodeling in Ulcerative Colitis,” Journal of Neurochemistry 168 (2024): 1937–1955, 10.1111/jnc.16080. 38426587

[18] M. D. Hu and K. L. Edelblum , “Sentinels at the Frontline: The Role of Intraepithelial Lymphocytes in Inflammatory Bowel Disease,” Current Pharmacology Reports 3 (2017): 321–334, 10.1007/s40495-017-0105-2. 29242771 PMC5724577

[19] M. J. Hamilton , S. M. Frei , and R. L. Stevens , “The Multifaceted Mast Cell in Inflammatory Bowel Disease,” Inflammatory Bowel Diseases 20 (2014): 2364–2378, 10.1097/MIB.0000000000000142. 25401721 PMC4428674

[20] M. D. Hu , A. D. Ethridge , R. Lipstein , et al., “Epithelial IL‐15 Is a Critical Regulator of γδ Intraepithelial Lymphocyte Motility Within the Intestinal Mucosa,” Journal of Immunology 201 (2018): 747–756, 10.4049/jimmunol.1701603. PMC6075741 29884699

[21] G. Boeckxstaens , “Mast Cells and Inflammatory Bowel Disease,” Current Opinion in Pharmacology 25 (2015): 45–49, 10.1016/j.coph.2015.11.005. 26629596

[22] S. Ben‐Horin and Y. Chowers , “Neuroimmunology of the Gut: Physiology, Pathology, and Pharmacology,” Current Opinion in Pharmacology 8 (2008): 490–495, 10.1016/j.coph.2008.07.010. 18675937

[23] A. C. Petrey and C. A. de la Motte , “The Extracellular Matrix in IBD: A Dynamic Mediator of Inflammation,” Current Opinion in Gastroenterology 33 (2017): 234–238, 10.1097/MOG.0000000000000368. 28562487 PMC5562400

[24] P. Pakshir and B. Hinz , “The Big Five in Fibrosis: Macrophages, Myofibroblasts, Matrix, Mechanics, and Miscommunication,” Matrix Biology 68–69 (2018): 81–93, 10.1016/j.matbio.2018.01.019. 29408013

[25] K. Y. Abboud , B. B. da Luz , J. L. Dallazen , et al., “Gastroprotective Effect of Soluble Dietary Fibres From Yellow Passion Fruit (*Passiflora edulis* f. *flavicarpa*) Peel Against Ethanol‐Induced Ulcer in Rats,” Journal of Functional Foods 54 (2019): 552–558, 10.1016/j.jff.2019.02.003.

[26] T. M. C. C. Filisetti‐Cozzi and N. C. Carpita , “Measurement of Uronic Acids Without Interference From Neutral Sugars,” Analytical Biochemistry 197 (1991): 157–162, 10.1016/0003-2697(91)90372-Z. 1952059

[27] A. R. Trevizan , S. L. Vicentino‐Vieira , P. da Silva Watanabe , et al., “Kinetics of Acute Infection With *Toxoplasma gondii* and Histopathological Changes in the Duodenum of Rats,” Experimental Parasitology 165 (2016): 22–29, 10.1016/J.EXPPARA.2016.03.015. 26993084

[28] T. Boeing , M. B. Gois , P. de Souza , L. B. Somensi , D. de M.G. Sant'Ana , and L. M. da Silva , “Irinotecan‐Induced Intestinal Mucositis in Mice: A Histopathological Study,” Cancer Chemotherapy and Pharmacology 87, no. 2021 (2021): 327–336, 10.1007/s00280-020-04186-x. 33130913

[29] M. J. Pastre , L. Casagrande , M. B. Gois , et al., “ *Toxoplasma gondii* Causes Increased ICAM‐1 and Serotonin Expression in the Jejunum of Rats 12 h After Infection,” Biomedicine & Pharmacotherapy 114 (2019): 108797, 10.1016/j.biopha.2019.108797. 30951950

[30] D. M. G. Sant'Ana , M. B. Gois , J. N. Zanoni , A. V. da Silva , C. J. T. da Silva , and E. J. A. Araújo , “Intraepithelial Lymphocytes, Goblet Cells and VIP‐IR Submucosal Neurons of Jejunum Rats Infected With *Toxoplasma gondii* ,” International Journal of Experimental Pathology 93, no. 2 (2012): 279–286, 10.1111/j.1365-2613.2012.00824.x. 22804764 PMC3444984

[31] M. J. Pastre , M. B. Gois , L. Casagrande , et al., “Acute Infection With *Toxoplasma gondii* Oocysts Preferentially Activates Non‐Neuronal Cells Expressing Serotonin in the Jejunum of Rats,” Life Sciences 283 (2021): 119872, 10.1016/j.lfs.2021.119872. 34352261

[32] A. Aguiar , A. S. S. Menezes de Brito , A. G. A. dos Santos , et al., “Mastocytosis and Intraepithelial Lymphocytosis in the Ileum and Colon Characterize Chronic *Toxoplasma gondii* Infection in Mice,” Tissue & Cell 91 (2024): 102533, 10.1016/j.tice.2024.102533. 39213782

[33] D. E. V. Pires , D. B. Ascher , and T. L. Blundell , “mCSM: Predicting the Effects of Mutations in Proteins Using Graph‐Based Signatures,” Bioinformatics 30 (2014): 335–342, 10.1093/bioinformatics/btt691. 24281696 PMC3904523

[34] J. A. Rocha , N. C. S. Rego , B. T. S. Carvalho , et al., “Computational Quantum Chemistry, Molecular Docking, and ADMET Predictions of Imidazole Alkaloids of Pilocarpus Microphyllus With Schistosomicidal Properties,” PLoS One 13 (2018): e0198476, 10.1371/journal.pone.0198476. 29944674 PMC6019389

[35] H. M. Berman , J. Westbrook , Z. Feng , et al., “The Protein Data Bank,” Nucleic Acids Research 28 (2000): 235–242, 10.1093/nar/28.1.235. 10592235 PMC102472

[36] Y. Li , A. S. Fanning , J. M. Anderson , and A. Lavie , “Structure of the Conserved Cytoplasmic C‐Terminal Domain of Occludin: Identification of the ZO‐1 Binding Surface,” Journal of Molecular Biology 352 (2005): 151–164, 10.1016/j.jmb.2005.07.017. 16081103

[37] M. A. Lee , L. Tan , H. Yang , Y.‐G. Im , and Y. J. Im , “Structures of PPARγ Complexed With Lobeglitazone and Pioglitazone Reveal Key Determinants for the Recognition of Antidiabetic Drugs,” Scientific Reports 7 (2017): 16837, 10.1038/s41598-017-17082-x. 29203903 PMC5715099

[38] M. E. Noguera , J. Jakoncic , and M. R. Ermácora , “High‐Resolution Structure of Intramolecularly Proteolyzed Human Mucin‐1 SEA Domain,” Biochimica et Biophysica Acta. Proteins and Proteomics 1868 (2020): 140361, 10.1016/j.bbapap.2020.140361. 31923589

[39] G. Javitt , L. Khmelnitsky , L. Albert , et al., “Assembly Mechanism of Mucin and von Willebrand Factor Polymers,” Cell 183 (2020): 717–729.e16, 10.1016/j.cell.2020.09.021. 33031746 PMC7599080

[40] D. S. Goodsell , G. M. Morris , and A. J. Olson , “Automated Docking of Flexible Ligands: Applications of AutoDock,” Journal of Molecular Recognition 9 (1996): 1–5, 10.1002/(sici)1099-1352(199601)9:1<1::aid-jmr241>3.0.co;2-6. 8723313

[41] G. M. Morris , R. Huey , and A. J. Olson , “Using AutoDock for Ligand‐Receptor Docking,” in Current Protocols in Bioinformatics (Wiley Interscience, 2008) Chapter 8. Unit 8.14., 10.1002/0471250953.bi0814s24. 19085980

[42] J. Gasteiger and M. Marsili , “Iterative Partial Equalization of Orbital Electronegativity—A Rapid Access to Atomic Charges,” Tetrahedron 36 (1980): 3219–3228, 10.1016/0040-4020(80)80168-2.

[43] D. Kozakov , L. E. Grove , D. R. Hall , et al., “The FTMap Family of Web Servers for Determining and Characterizing Ligand‐Binding Hot Spots of Proteins,” Nature Protocols 10 (2015): 733–755, 10.1038/nprot.2015.043. 25855957 PMC4762777

[44] G. M. Morris , D. S. Goodsell , R. S. Halliday , et al., “Automated Docking Using a Lamarckian Genetic Algorithm and an Empirical Binding Free Energy Function,” Journal of Computational Chemistry 19 (1998): 1639–1662, 10.1002/(SICI)1096-987X(19981115)19:14<1639::AID-JCC10>3.0.CO;2-B.

[45] F. J. Solis and R. J.‐B. Wets , “Minimization by Random Search Techniques,” Mathematics of Operations Research 6 (1981): 19–30, http://www.jstor.org/stable/3689263.

[46] J. H. Lin and M. Yamazaki , “Role of P‐Glycoprotein in Pharmacokinetics: Clinical Implications,” Clinical Pharmacokinetics 42 (2003): 59–98, 10.2165/00003088-200342010-00003. 12489979

[47] P. B. Burns , R. J. Rohrich , and K. C. Chung , “The Levels of Evidence and Their Role in Evidence‐Based Medicine,” Plastic and Reconstructive Surgery 128 (2011): 305–310, 10.1097/PRS.0b013e318219c171. 21701348 PMC3124652

[48] X. Ma , C. Chen , and J. Yang , “Predictive Model of Blood‐Brain Barrier Penetration of Organic Compounds,” Acta Pharmacologica Sinica 26 (2005): 500–512, 10.1111/j.1745-7254.2005.00068.x. 15780201

[49] C. M. Bowman and L. Z. Benet , “An Examination of Protein Binding and Protein‐Facilitated Uptake Relating to In Vitro‐In Vivo Extrapolation,” European Journal of Pharmaceutical Sciences 123 (2018): 502–514, 10.1016/j.ejps.2018.08.008. 30098391 PMC6365006

[50] J. Chen , H. Yang , L. Zhu , et al., “In Silico Prediction of Human Renal Clearance of Compounds Using Quantitative Structure‐Pharmacokinetic Relationship Models,” Chemical Research in Toxicology 33 (2020): 640–650, 10.1021/acs.chemrestox.9b00447. 31957435

[51] T. E. Archer , “Acute Oral Toxicity as LD50 (mg/kg) of Propargyl Alcohol to Male and Female Rats,” Journal of Environmental Science and Health, Part B 20 (1985): 593–596, 10.1080/03601238509372497. 4078233

[52] D. E. V. Pires , T. L. Blundell , and D. B. Ascher , “pkCSM: Predicting Small‐Molecule Pharmacokinetic and Toxicity Properties Using Graph‐Based Signatures,” Journal of Medicinal Chemistry 58 (2015): 4066–4072, 10.1021/acs.jmedchem.5b00104. 25860834 PMC4434528

[53] I. Okayasu , S. Hatakeyama , M. Yamada , T. Ohkusa , Y. Inagaki , and R. Nakaya , “A Novel Method in the Induction of Reliable Experimental Acute and Chronic Ulcerative Colitis in Mice,” Gastroenterology 98 (1990): 694–702, 10.1016/0016-5085(90)90290-h. 1688816

[54] N. M. T. de Oliveira , B. Barbosa da Luz , V. S. Schneider , et al., “Dietary Polysaccharides From Guavira Pomace, a Co‐Product From the Fruit Pulp Industry, Display Therapeutic Application in Gut Disorders,” Food Research International 156 (2022): 111291, 10.1016/j.foodres.2022.111291. 35651057

[55] D. Maria‐Ferreira , L. M. Da Silva , D. A. G. B. Mendes , et al., “Rhamnogalacturonan From *Acmella oleracea* (L.) R.K. Jansen: Gastroprotective and Ulcer Healing Properties in Rats,” PLoS One 9 (2014): e84762, 10.1371/JOURNAL.PONE.0084762. 24416280 PMC3885607

[56] W. Lai , Y. Wang , C. Huang , et al., “DIREN Mitigates DSS‐Induced Colitis in Mice and Attenuates Collagen Deposition via Inhibiting the Wnt/β‐Catenin and Focal Adhesion Pathways,” Biomedicine & Pharmacotherapy 175 (2024): 116671, 10.1016/j.biopha.2024.116671. 38678963

[57] U. Erben , C. Loddenkemper , K. Doerfel , et al., “A Guide to Histomorphological Evaluation of Intestinal Inflammation in Mouse Models,” International Journal of Clinical and Experimental Pathology 7 (2014): 4557–4576. 25197329 PMC4152019

[58] S. H. Lee , “Intestinal Permeability Regulation by Tight Junction: Implication on Inflammatory Bowel Diseases,” Intestinal Research 13 (2015): 11–18, 10.5217/ir.2015.13.1.11. 25691839 PMC4316216

[59] J. B. Furness , “The Enteric Nervous System and Neurogastroenterology,” Nature Reviews. Gastroenterology & Hepatology 9 (2012): 286–294, 10.1038/nrgastro.2012.32. 22392290

[60] O. W. Meldrum , G. E. Yakubov , G. Gartaula , M. A. McGuckin , and M. J. Gidley , “Mucoadhesive Functionality of Cell Wall Structures From Fruits and Grains: Electrostatic and Polymer Network Interactions Mediated by Soluble Dietary Polysaccharides,” Scientific Reports 7 (2017): 15794, 10.1038/s41598-017-16090-1. 29150632 PMC5694006

